# COM5/443: Health Portals, Search Engines and Intranets: Making order of the chaos - A review article

**DOI:** 10.2196/jmir.1.suppl1.e18

**Published:** 1999-09-19

**Authors:** P Maini

**Affiliations:** ^1^Radical Technology, LondonUK

**Keywords:** Health Portals

## Abstract

Effective use of technology has led to medical advances that have not only extended life expectancy, but also fuelled an increasingly well informed public's desires to expect more and more from today's healthcare providers. A phenomenal amount of medical information is now present, facilitated by the virtually unrestricted ability to publish on the Internet. The WWW grows by roughly a million pages per day with a significant number devoted to some element of healthcare. As a consequence of the web's rapid, chaotic growth, the resulting network of information lacks organisation and structure and the quest for a method of finding relevant and reliable information quickly is spawning the growth of the Internet Portal Sites. The use of intranets confers some degree of organisation, allowing for structured growth of an organisation's web site but the majority of sites are not sheltered within an intranet. Millions of dollars are being invested in researching the technologies powering search engines and well designed portals, in the hope of imposing some kind of order on the existing chaos. The winner will most likely reap huge financial rewards as advertisers climb on board, hoping to gain massive exposure and the public will undoubtedly gain from relevant and timely answers to their searches and queries.

Information is a valuable commodity, but the true value is seen only when it is organised into an accessible and useable form. The coming of age of the Internet has heralded radical changes in the fabric and economics of healthcare and as a consequence our expectations from professionals involved in this industry are greatly increased. Although significant challenges need to be overcome, there are huge benefits to be gained for consumers, although it remains to be seen whether the world becomes a more healthy place.

